# Low serum 25-hydroxyvitamin D levels and acute kidney injury in the critically ill

**DOI:** 10.1186/cc10758

**Published:** 2012-03-20

**Authors:** AB Braun, AA Litonjua, T Moromizato, FK Gibbons, E Giovannucci, KB Christopher

**Affiliations:** 1 Brigham and Women's Hospital, Boston, MA, USA; 2 Massachusetts General Hospital, Boston, MA, USA; 3 Harvard School of Public Health, Boston, MA, USA

## Introduction

Given the importance of inflammation in acute kidney injury and the relationship between vitamin D and inflammation, we sought to elucidate the effect of vitamin D status on acute kidney injury. We hypothesized that deficiency in 25-hydroxyvitamin D (25(OH)D) prior to hospital admission would be associated with acute kidney injury in the critically ill.

## Methods

We performed an observational study of patients treated in medical and surgical ICUs in two teaching hospitals in Boston, Massachusetts between 1998 and 2009. We studied 2,075 patients, age ≥18 years, in whom serum 25(OH)D was measured prior to hospitalization. The exposure of interest was pre-admission serum 25(OH)D and categorized *a priori *as deficiency (25(OH)D ≤15 ng/ml), insufficiency (25(OH)D 15 to 30 ng/ml) or sufficiency (25(OH)D ≥30 ng/ml). The primary outcome was acute kidney injury defined as meeting RIFLE Injury or Failure criteria in the 7 days prior to critical care initiation and the 7 days following critical care initiation. We applied the serum creatinine criteria to determine the maximum RIFLE class. Pre-admission baseline creatinine was available on all subjects. Logistic regression examined the RIFLE criteria outcome. Adjusted odds ratios (ORs) were estimated by multivariable logistic regression models. Estimates were adjusted for age, gender, race (white, nonwhite), Deyo-Charlson index, sepsis and patient type (surgical vs. medical).

## Results

Pre-admission 25(OH)D deficiency is predictive for acute kidney injury. Patients with 25(OH)D deficiency have an OR for acute kidney injury of 1.73 (95% CI, 1.30 to 2.30; *P *< 0.0001) relative to patients with 25(OH)D sufficiency. The 25(OH)D deficiency remains a significant predictor of acute kidney injury following multivariable adjustment (adjusted OR 1.50; 95% CI, 1.42 to 2.24; *P *< 0.0001). Patients with 25(OH) D insufficiency have an OR for acute kidney injury of 1.49 (95% CI, 1.15 to 1.94; *P *= 0.003) and an adjusted OR of 1.23 (95% CI, 1.12 to 1.72; *P *= 0.003) relative to patients with 25(OH)D sufficiency. The vitamin D-acute kidney injury association is independent of the time between 25(OH)D draw and hospital admission.

## Conclusion

Deficiency of 25(OH)D prior to hospital admission is a significant predictor of acute kidney injury in a critically ill patient population.

